# Administration frequency as well as dosage of PTH are associated with development of cortical porosity in ovariectomized rats

**DOI:** 10.1038/boneres.2017.2

**Published:** 2017-04-25

**Authors:** Aya Takakura, Ji-Won Lee, Kyoko Hirano, Yukihiro Isogai, Toshinori Ishizuya, Ryoko Takao-Kawabata, Tadahiro Iimura

**Affiliations:** 1 Pharmaceuticals Research Center, Asahi Kasei Pharma Corporation, Shizuoka, Japan; 2 Graduate School of Medicine, Division of Analytical Bio-Medicine, Ehime University, Ehime, Japan; 3 Division of Bio-Imaging, Proteo-Science Center (PROS), Ehime University, Ehime, Japan; 4 Division of Analytical Bio-Medicine, Advanced Research Support Center (ADRES), Ehime University, Ehime, Japan; 5 Artificial Joint Integrated Center, Ehime University Hospital, Ehime, Japan

## Abstract

To investigate whether the administration frequency of parathyroid hormone (PTH) is associated with the development of cortical porosity, this study established 15 dosage regimens of teriparatide [human PTH(1–34), TPTD] with four distinct concentrations and four distinct administration frequencies of TPTD to 16-week-old ovariectomized rats. Our analyses demonstrated that the bone mineral density, mechanical properties, and bone turnover were associated with the total amount of TPTD administered. Our observations further revealed that the cortical porosity was markedly developed as a result of an increased administration frequency with a lower concentration of total TPTD administration in our setting, although the highest concentration also induced cortical porosity. Deconvolution fluorescence tiling imaging on calcein-labeled undecalcified bone sections also demonstrated the development of cortical porosity to be closely associated with the bone site where periosteal bone formation took place. This site-specific cortical porosity involved intracortical bone resorption and an increased number and proximity of osteocytic lacunae, occasionally causing fused lacunae. Taken together, these findings suggested the involvement of local distinctions in the rate of bone growth that may be related to the site-specific mechanical properties in the development of cortical porosity induced by frequent and/or high doses of TPTD.

## Introduction

The effects of parathyroid hormone (PTH) on rat bone metabolism were first observed in the early 1900s.^1–3^ Since the achievement of the chemical synthesis of human PTH(1–34) in the 1970s, studies have attempted to define the detailed mechanism of this hormonal action.^4^ The anabolic effect of PTH on the skeleton in post-menopausal osteoporosis reported by Reeve *et al*^5^ was the first clinical application.

PTH is well known to exert its anabolic effects on bone through promoting the proliferation, differentiation and survival of bone-forming osteoblasts, which concomitantly induce the differentiation of osteoclasts, thereby promoting bone resorption.^6^ The balance between these anabolic and catabolic actions of PTH administration is thought to be defined by the blood concentration of PTH, although the exact molecular and cellular mechanisms underlying these phenomena remain controversial. Bone formation and resorption are preferentially promoted when blood PTH level are intermittently and persistently high, respectively. In other words, the effect of PTH on bone is dosage regimen-dependent. The intermittent exposure of PTH increases bone formation over resorption, whereas the continuous infusion of PTH stimulates bone resorption over formation, causing a net loss in bone mass.^7,8^

PTH binds to its cell surface receptor PTH1R, which is expressed in osteoblasts and their precursors, chondrocytes, and vascular smooth muscle cells. Recent findings have demonstrated that PTH1R-mediated signaling causes two distinct processes: a canonical G protein-coupled receptor (GPCR)-cyclic AMP (cAMP) pathway and an endocytosis-mediated pathway.^9^ The former induces a transient cAMP response derived from signaling complexes localized at the plasma membrane, whereas the latter induces a prolonged cAMP response mediated by molecular complexes associated within the endosome. Studies using PTH analogs *in vivo* have suggested that selective stimulation of the endosome-mediated long signal and the prolonged half-life of PTH in the blood are favorable for inducing hypercalcemia.^10,11^ However, the association between the temporal variations in PTH-mediated cAMP signaling and the downstream cellular responses that promote bone anabolism and catabolism remains unclear.

Cortical porosity occurs mainly by the stimulation of intracortical remodeling, thus causing thinner cortices, as clinically observed in elderly women and women with hyperparathyroidism.^12–14^ Recent findings have emphasized the relevance of cortical porosity as a determinant of bone fragility that is highly associated with fracture risk, although clinical and basic studies have tended to focus on vertebral fractures and trabecular bone loss.^15–18^ The continuous stimulation of PTH has been reported to induce the early development of cortical porosity.^19^ Once-daily administration of teriparatide [human PTH(1–34), TPTD] has been found to increase cortical porosity in clinical studies^20^ and animal studies using rabbits^21^ and dogs.^22^ These findings suggest that the dosage regimen of PTH affects not only the net bone mass but also the microarchitecture of bone, including cortical porosity, which may in turn affect the pharmacological action of PTH with respect to preventing the risk of bone fracture. Members of our research group have recently reported that a long-term and lower frequency (three times weekly) TPTD administration in ovariectomized (OVX) rats improves the bone density, microarchitecture, and mechanical properties of bone without obvious stimulation of bone resorption.^23^ However, there is little information in the literature concerning the development of cortical porosity at any particular frequency or dosage of PTH administration, although such information would provide relevant information to clinical settings involving PTH treatment.

In this study, we established 15 regimens with four distinct concentrations and four distinct administration frequencies of TPTD administration to 13-week-old OVX rats as a standard post-menopausal osteoporosis animal model for PTH examination^23–25^ and then analyzed the bone properties, focusing on the development of cortical porosity.

## Materials and methods

### Animals

Eleven-week-old, sexually mature female Sprague-Dawley rats (Charles River, Kanagawa, Japan) were used. The rats were maintained under a 12-h light/dark cycle and given unrestricted access to tap water and a standard diet containing 1.2% calcium, 0.9% phosphorus, 22.0% protein, and 6.2 IU vitamin D3 per gram (CRF-1; Oriental Yeast, Tokyo, Japan). The animals were allowed to acclimate to their environment for 2 weeks before the start of the experiment. The experimental protocols were approved by the experimental animal ethics committee at Asahi Kasei Pharma Corporation and were conducted in accordance with the guidelines concerning the management and handling of experimental animals.

### Experimental design

At 13 weeks of age, sexually mature rats were divided into 15 groups (Table 1) and subjected to either bilateral ovariectomy or sham surgery under anesthesia comprising intraperitoneal injection with ~48 mg·kg^−1^ ketamine hydrochloride and 4 mg·kg^−1^ xylazine, as previously described.^23,26^ For the sham surgery, ~10-mm-long incisions were made on the back of each rat, as in the ovariectomy, and the ovaries were exposed and then replaced.

Three weeks after the surgery, at 16 weeks of age, the rats were subcutaneously injected with 6 or 30 μg·kg^−1^ of chemically synthetized TPTD acetate (Asahi Kasei Pharma Corporation, Tokyo, Japan) three times a week (W3 groups); 1.2, 6 or 30 μg·kg^−1^ of TPTD once a day (D1 groups) or twice a day (D2 groups); and 1.2 or 6 μg·kg^−1^ of TPTD three times a day (D3 groups) for 4 weeks. Saline injection was given to the sham animals (three times a week) and each group as the vehicle. In total, 15 experimental groups were established and comparatively analyzed.

A double-fluorochrome labeling technique was used to determine the active mineralization sites and rates of bone formation. Calcein (Dojindo Laboratories, Kumamoto, Japan) was subcutaneously injected into each rat twice at a dose of 10 mg·kg^−1^ body weight on the 8th and 3rd days before killing. This regimen was selected after considering the effects of aging and OVX and to appropriately measure the fluorochrome-based parameters.^27^ After the dosing period, the rats were killed under anesthesia, and the fourth and fifth lumbar vertebrae (LV4 and LV5) were collected. There were no deaths during surgery. The rats awoke from anesthesia ~3 h after surgery and began feeding. There were no clear behavioral differences after surgery between the OVX- and sham-operated groups. The uterine weight at necropsy revealed uterine atrophy in all rats in the OVX group, thus confirming that the procedure had been performed correctly.

### Measurement of bone metabolic markers

**Figure 1:**
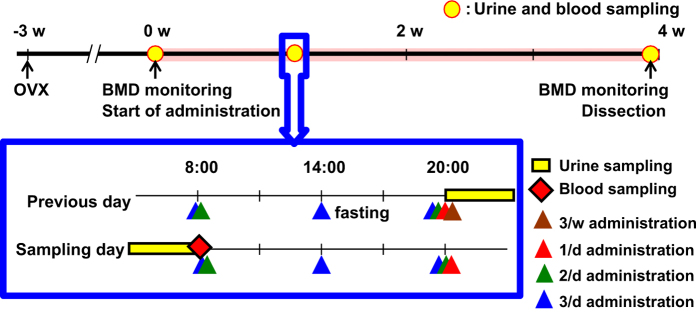
Time course of TPTD administration and sampling. Rats at 13 weeks of age were subjected to either ovariectomy or sham surgery. Three weeks after the surgery, TPTD administration was started. The serum and urine were collected at pre-dosing (day 0), the 9th day (day 9), and the 28th day (day 28) after the initial administration.

The serum and urine were collected at pre-dosing (day 0), the 9th day (day 9) and the 28th day (day 28) after the initial administration (Figure 1). All rats were fasted for at least 6 h before blood and urine collection, and were then placed in metabolic cages without food for 12 h. The serum and urine samples were stored at −80 °C until analysis. The time schedules for fasting, urine and blood sampling are also shown in Figure 1.

The urinary level of C-terminal telopeptide of type I collagen (CTX), a bone resorption marker, was measured using a RatLaps EIA (Immunodiagnostic Systems, Boldon, UK). The urinary level of creatinine was measured using L-type Wako CRE·M (Wako Pure Chemical Industries, Osaka, Japan). The urinary level of CTX was corrected for the urinary level of creatinine. The serum level of intact osteocalcin (OC), a bone formation marker, was measured using an OC rat ELISA system (GE Healthcare Bioscience, Tokyo, Japan). All the assays were performed in accordance with the manufacturers’ instructions.

### Preparation of bone samples

The LV4 was resected at the vertebral arch and the transverse and spinous processes. A central cylindrical specimen with parallel ends and a mean height±s.d. of 3.7±0.1 mm was obtained from each vertebral body for further measurements by using a diamond band saw (BS-3000; Exakt, Norderstedt, Germany).

The LV5 was removed and dissected free of soft tissue, fixed in 70% ethanol, stained with Villanueva bone stain, dehydrated in a graded series of ethanol, defatted in acetone and embedded in polymethyl methacrylate (Wako Pure Chemical Industries). Thin sections (5 μm) were prepared from a sagittal section of the central LV5. In addition, thin ground sections were prepared from a cross-section of the central LV5 with the remaining blocks.

### Measurement of the bone mineral density

The bone mineral density (BMD) of the LV4 vertebral cylinders was measured using dual-energy X-ray absorptiometry (DCS-600EX-3R; Aloka, Tokyo, Japan). The specimens were placed on the scanning table ventral side up and scanned at a pitch of 1 mm and speed of 25 mm·min^−1^. The BMD (mg·cm^−2^) was calculated from the bone mineral content (mg) and bone area (cm^2^).

### Micro structure analyses for the cortical bone of LV4

A cone-beam X-ray micro-computed tomography (CT) system (ScanXmate-RB090SS150; Comscantecno, Kanagawa, Japan) was used to obtain CT images of the isolated bone samples using the following settings: tube voltage, 70 kV; tube current, 0.1 mA; and voxel size, 12.5×12.5×12.5 μm^3^. Three-dimensional images were reconstructed and analyzed using the TRI/3D-BON software program (RATOC System Engineering, Tokyo, Japan). We analyzed the middle portion (height, 2 mm; 160 slices) of the lumbar vertebral cylinder. The images were binarized with a fixed threshold (404 mg·cm^−3^ converted as BMD). Spatial dissociation of the cortical and trabecular areas was defined by lengths and angles of trabeculae protruding from the inner periosteum according to the manufacturer’s instructions.

The cortical bone structure was determined in the ventral cortical bone of LV4 and the following parameters were measured: cortical bone volume (Cv, mm^3^) and cortical void volume (Vv/Cv, %).

### Mechanical properties of LV4

Compression testing of the vertebral body was performed as previously described.^28^ The vertebral cylinder specimens of LV4 were placed on a lower platen, cranial-side up and compressed with a 4-mm-diameter upper platen with a material testing machine (EZ-L-1kN; Shimadzu, Tokyo, Japan) at a constant speed of 2 mm·min^−1^. The load and displacement curves were recorded, and the following extrinsic parameters were calculated using the testing machine’s software program (TRAPEZIUM2; Shimadzu): maximum load (N), stiffness (N·mm^−1^), and breaking energy (hereafter referred to as energy; N·mm).

### Bone histomorphometry and microscopy

The bone histomorphometric parameters related to bone mass, structure, resorption, formation, and turnover were measured using an image analysis system (Histometry RT Camera, System Supply, Nagano, Japan; OsteoMeasure, Decatur, GA, USA). The nomenclature, symbols, and units used in the present study are those recommended by the Nomenclature Committee of the American Society for Bone and Mineral Research.^29,30^ Histomorphometric measurements were obtained from cancellous bone tissue in the secondary spongiosa region of the sagittal sections of LV5.

The following static parameters were measured: trabecular bone volume (BV/TV), trabecular thickness (Tb.Th) and trabecular number (Tb.N). The following bone resorption parameters were measured: eroded surface (ES/BS) and osteoclast surface (Oc.S/BS). The following bone formation and turnover parameters were measured: osteoid surface (OS/BS), mineralizing surface (MS/BS), osteoblast surface (Ob.S/BS, based on the double plus half single label), bone formation rate (BFR/BV), and activation frequency (Ac.f).^29,30^

To analyze the cortical porosity, horizontal sections of LV4 were analyzed using a semi-automated system (OsteoMeasure). The BV/TV, cortical porosity, and osteocyte number (No.Ot/B.Ar) were scored separately from the dorsal and ventral cortical walls and the middle of the trabecular area of the LV4 vertebral body. Small porosities, osteocytic lacunae, and fused lacunae were morphologically dissociated in these scoring in which protrusion and connection of osteocytic canaliculi was the most prominent morphological land mark for this distinction.

Bright-field and fluorescence images of the calcein-labeled undecalcified bone sections were obtained by a microscopy system, ECLIPSE Ni (Nikon, Tokyo, Japan) equipped with differential interference contrast microscopy and objectives (Nikon), as follows: Plan Apo λ ×10 (numerical aperture (NA)=0.45), Plan Apo λ ×20 (NA=0.75), and Plan Apo λ ×40 (NA=0.95). The fluorescence signals were obtained using a filter sets, GFP-B (excitation: 460–500 nm, DM: 505 nm, emission: 510–560 nm; Nikon) for calcein and TxRed (excitation: 540–580 nm, DM: 595 nm, emission: 600–660 nm; Nikon) for auto-fluorescence derived from soft tissue. Tiling fluorescence imaging to acquire the entire, high-contrast view of the tissue sections was carried out using a Plan Apo λ ×10 objective (NA=0.45). The frame size of a single scan was 1 280×1 024 pixels with an 8-bit color depth. The fluorescence and differential interference contrast images were sequentially acquired, with a pixel size of 0.64 μm. A total of 4×7 images were combined for tiling. Image processing, including deconvolution, was performed using the imaging software program NIS-elements AR (Nikon).

### Statistical analyses

All the data are presented as mean±s.d. The effects of TPTD were investigated using an analysis of variance. The variables showing a difference between the TPTD treatment groups and the vehicle control group of each administration frequency were then analyzed with Dunnett’s test (*post hoc*). Statistical significance was defined as *P*<0.05.

## Results

### Effects of TPTD administration regimen in OVX rats on BMD, mechanical properties, and systemic bone markers

To analyze the effect of the TPTD administration frequency on bone quality in OVX rats, we established four administration schedules: three times a week (3/w), once a day (1/d), twice a day (2/d), and three times a day (3/d) with four distinct TPTD doses: 0 (vehicle: V), 1.2 (low: L), 6 (medium: M), and 30 μg·kg^−1^ (high: H) for 4 weeks (Table 1).

**Figure 2:**
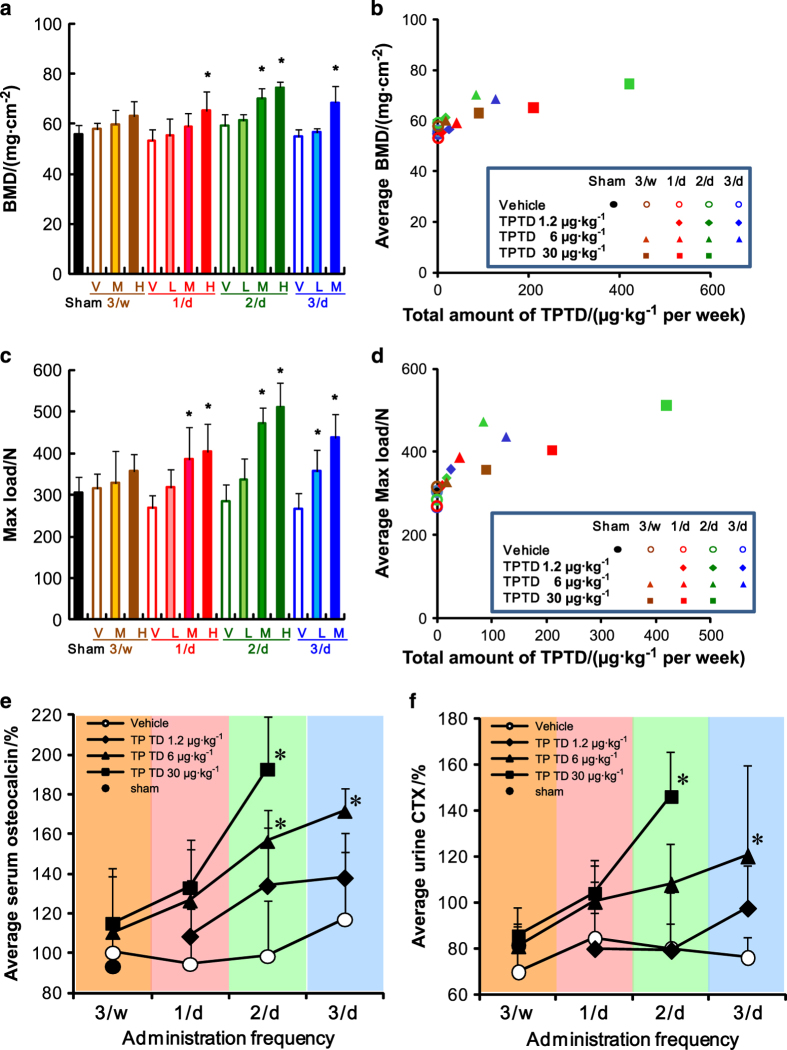
The bone mineral density and mechanical strength of the 4th lumbar vertebral body, and changes in the bone metabolic markers in serum and urine. (**a**, **b**) BMD of LV4 vertebral body measured by dual-energy X-ray absorptiometry is plotted against the administration regimen; (**a**) and total amount of TPTD administered (μg·kg^−1^ per week; **b**). (**c**, **d**) The mechanical properties of the LV4 vertebral body as measured by a compression test are plotted against the administration regimen (**c**) and the total amount of TPTD (μg·kg^−1^ per week) administered (**d**). (**a** and **c**) The data are shown as the means+s.d. (*n*=4 or 5). * Indicates *P*<0.05 vs vehicle of each administration frequency (analysis of variance (ANOVA) with *post hoc* Dunnett’s tests). V: vehicle control, L: 1.2 μg·kg^−1^, M: 6 μg·kg^−1^, H: 30 μg·kg^−1^. (**b**, **d**) The data are shown as the means (*n*=4 or 5). Black closed circle: Sham control. Blue: three times a day, green: two times a day, red: once a day, brown: three times a week. Opened circle: vehicle control, diamond: 1.2 μg·kg^−1^, triangle: 6 μg·kg^−1^, square: 30 μg·kg^−1^. (**e**, **f**) Serum osteocalcin level (**e**) and urine CTX level (**f**) at day 9 are plotted against the administration frequency of TPTD. (**e**, **f**) The data are shown as the means (*n*=4 or 5). * Indicates *P*<0.05 vs vehicle of each administration frequency (two-way repeated measure ANOVA with *post hoc* Dunnett’s test). Black closed circle: Sham control, opened circle: vehicle control, diamond: 1.2 μg·kg^−1^, triangle: 6 μg·kg^−1^, square: 30 μg·kg^−1^.

We first analyzed the BMD of LV4 explants by using dual-energy X-ray absorptiometry (Figure 2a). In each group of distinct administration frequency, the BMD was obviously increased in a dose-dependent manner. However, in comparing the groups that received the same TPTD dose at different administration frequencies, we found that an increased administration frequency didn’t necessarily augment the BMD. In particular, when the effects on BMD in the 3/d and 2/d groups were compared, the effect in the 3/d group was found to be saturated. An analysis of the association of the average BMD and total amount of TPTD administered (μg·kg^−1^ per week; Figure 2b) showed that the BMD increased in a linear manner, especially at total doses <100 μg·kg^−1^ per week. However, in the groups that received ⩾100 μg·kg^−1^ per week, the effect on BMD appeared to be saturated.

We next analyzed the mechanical properties of the LV4 vertebral body by using the compression tests. As with the BMD, the average maximal loads of LV4 increased in a dose-dependent manner (Figure 2c). However, although the increase in the administration frequency for each TPTD dose also effectively augmented the mechanical properties, the effects of the highest frequency of 3/d on these properties were comparable to those in the 2/d group (Figure 2c). When the average mechanical properties of each regimen were plotted against the total amount of TPTD (μg·kg^−1^ per week) (Figure 2d), the effect of TPTD appeared to be saturated at doses⩾100 μg·kg^−1^ per week.

We also examined the trabecular bone in LV5 by using histomorphological approaches to further evaluate the effect of TPTD on bone metabolism in our regimen settings (Supplementary Figures 1 and 2). As was observed with BMD, BV/TV and Tb.Th were increased in a dose-dependent manner in each frequency group, and these parameters in the 3/d group were comparable to those in the 2/d group (Supplementary Figure 1A and B), although Tb.N did not show significant differences among any of the regimens (Supplementary Figure 1C). Bone formation parameters such as OS/BS, MS/BS, and Ob.S/BS showed similar tendencies to BV/TV and Tb.Th (Supplementary Figure 1D–F). Of note, 30 μg·kg^−1^ at 2/d (H: 2/d) did not markedly augment these parameters compared to 6 μg·kg^−1^ at 2/d (M: 2/d), indicating the saturation of the effects of TPTD administration on bone formation.

The bone resorption parameters such as ES/BS and Oc.S/BS did not show significant differences from the control group in any of the regimens; however, 1.2 μg·kg^−1^ at 3/d (L: 3/d) and 6 μg·kg^−1^ at 3/d (M: 3/d) augmented these bone resorption parameters, albeit not significantly (Supplementary Figure 2A and B). As with other turnover parameters, BFR/BV was significantly augmented by TPTD administration regimens, except for 6 μg·kg^−1^ at 3/w (M: 3/W) and 1.2 μg·kg^−1^ at 1/d (L: 1/d), whereas Ac.f exhibited similar patterns to the bone formation parameters (Supplementary Figure 2C and D).

To investigate the effects on systemic bone turnover, the changes in the levels of serum OC and urine CTX were examined as markers of bone formation and resorption, respectively (Figure 2e, f and Supplementary Figure 3A–D). The change in the rates of the average serum OC level were markedly increased compared to that of the vehicle control in each frequency group in a dose-dependent manner, although significant changes were observed only for 6 μg·kg^−1^ at 2/d (M: 2/D), 30 μg·kg^−1^ at 2/d (H: 2/D), and 1.2 μg·kg^−1^ at 3/d (M: 3/d; Figure 2e). Furthermore, when the same doses of TPTD in the distinct frequency groups were compared, the changes in the OC levels also increased in a frequency-dependent manner (Figure 2e).

The effects of the TPTD regimens on the average urine CTX showed distinct patterns from those on the average serum OC levels (Figure 2f and Supplementary Figure 3E–H). At the lowest dose of 1.2 μg·kg^−1^, only the CTX level in the highest frequency of 3/d (L: 3/d) was increased, albeit not significantly; in contrast, in the other frequency groups, the effects of 1.2 μg·kg^−1^ of TPTD administration (L: 1/d and L: 2/d) on the CTX level were comparable to those of the vehicle controls, demonstrating an obvious contrast in the effects of the different regimens on the OC levels. Significant increases in the CTX levels by TPTD administration were observed only in groups receiving 30 μg·kg^−1^ at 2/d (H: 2/D) and 1.2 μg·kg^−1^ at 3/d (M: 3/d). Therefore, the augmented effect on the CTX level by TPTD is only achieved at a higher administration frequency and higher dose than those of the effects on the OC level. Serum Ca levels in any settings did not show significant differences (data not shown).

### Spatial revelation of the development of cortical porosity depending on the frequency and dose of TPTD administration

**Figure 3:**
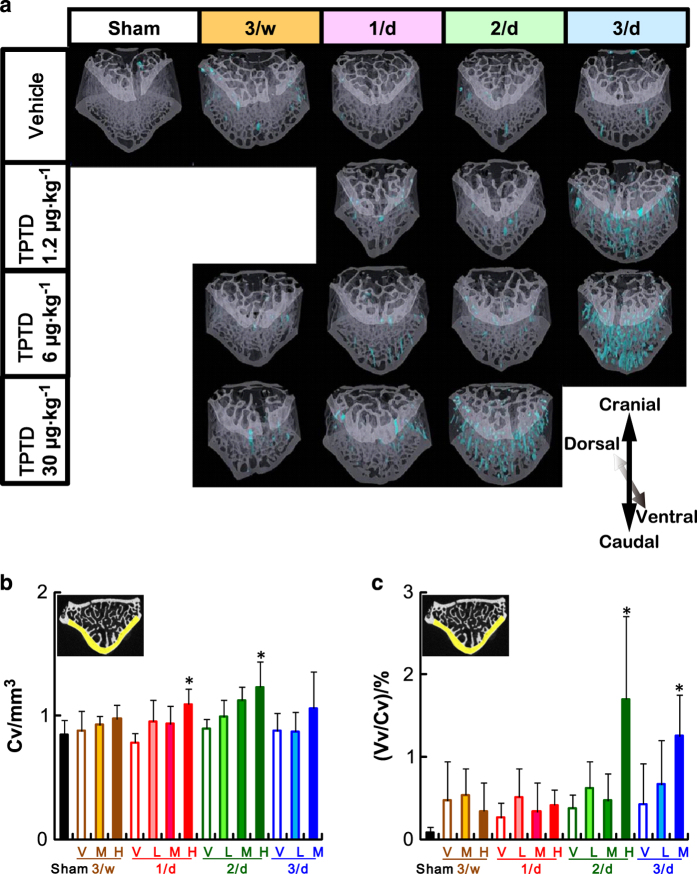
A micro-CT analysis of the cortical bone of LV4. (**a**) The three-dimensional reconstitution images of the bone structure of LV4. The region of cortical porosity is highlighted in blue. (**b**, **c**) Cortical bone volume (Cv) and (**b**) cortical void volume (Vv/Cv) (**c**) of ventral cortical bone of LV4 are plotted against the administration regimen of TPTD. The data are shown as the means+s.d. (*n*=4 or 5). * Indicates *P*<0.05 vs vehicle of each administration frequency (analysis of variance with *post hoc* Dunnett’s test). V: vehicle control, L: 1.2 μg·kg^−1^, M: 6 μg·kg^−1^, H: 30 μg·kg^−1^.

**Figure 4:**
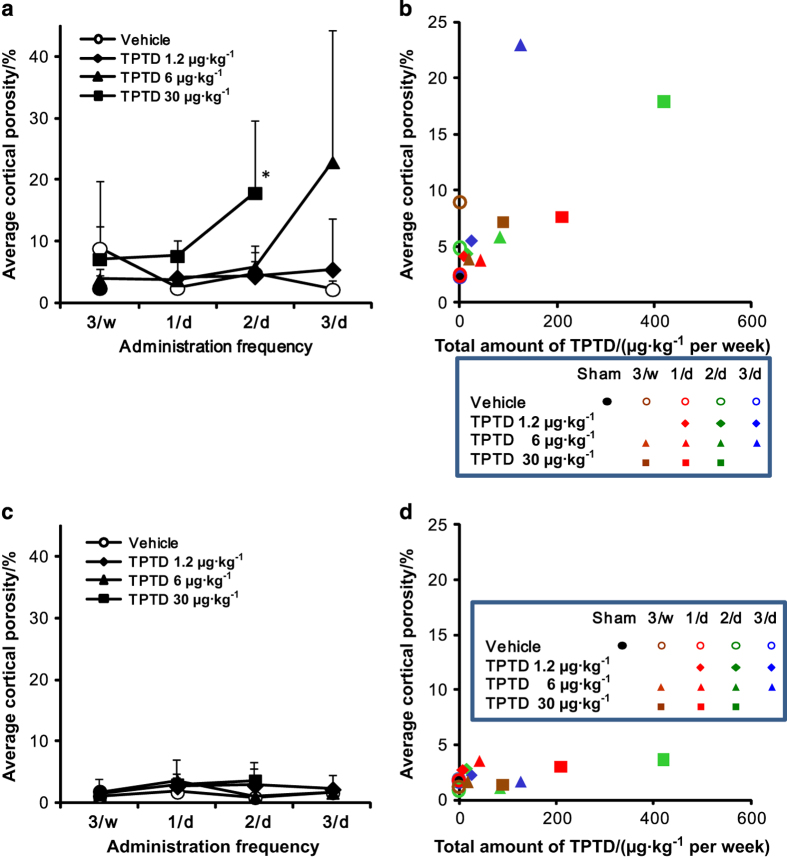
A comparison of the average cortical porosity between the ventral and dorsal portions of LV4. (**a**, **b**) The average cortical porosity in ventral wall of LV4 is plotted against the administration frequency (A) and total amount of TPTD administered (μg·kg^−1^ per week; **b**). (**c**, **d**) The average cortical porosity in the dorsal wall of LV4 is plotted against the administration frequency (**c**) and total amount of TPTD administered (μg·kg^−1^ per week; **d**). (**a**, **c**) The data are shown as the means (*n*=4 or 5). Black closed circle: Sham control, opened circle: vehicle control, diamond: 1.2 μg·kg^−1^, triangle: 6 μg·kg^−1^, square: 30 μg·kg^−1^. (**b**, **d**) Data represent means (*n*=4 or 5). Black closed circle: Sham control. Blue: three times a day, green: two times a day, red: once a day, brown: three times a week. Opened circle: vehicle control, diamond: 1.2 μg·kg^−1^, triangle: 6 μg·kg^−1^, square: 30 μg·kg^−1^.

We next assessed whether the administration frequency and/or dose of TPTD affects the cortical porosity, via micro-CT-based analyses. Whole-body scanning of specimens revealed that the vertebral bodies exhibited the most obvious cortical porosities in some regimen groups. We therefore focused our analyses on the lumbar vertebrae. Figure 4 shows three-dimensional reconstitution images of micro-CT scanning of LV4. Ventral views of a representative specimen in each regimen are arranged with the cortical porosity distribution area highlighted in blue. We observed increases in the distributions of cortical porosity, particularly in the ventral cortical bone, in the regimen groups of 30 μg·kg^−1^ at 2/d (H: 2/d) and 6 μg·kg^−1^ at 3/d (M: 3/d; Figure 3a). Significant increases in the cortical volume (Cv) were observed at 30 μg·kg^−1^ in the 1/d and 2/d groups (H: 1/d and H: 2/d; Figure 3b). In contrast, significant increases in the void volume/cortical volume (Vv/Cv) were observed in the groups receiving 30 μg·kg^−1^ at 2/d (H: 2/d) and 6 μg·kg^−1^ at 3/d (M: 3/d), suggesting that the appearance of cortical porosity is sensitive to the administration frequency as well as the total dose of TPTD (Figure 3c).

We next applied histomorphometric analyses to further analyze the cortical porosity caused by TPTD administration (Figure 4). Since our micro-CT-based observations indicated that cortical porosity was preferentially observed in the ventral cortical portion of LV4, the dorsal and ventral cortical bone were analyzed and compared. Histomorphometric analyses of the ventral cortical bone revealed obvious increases in the average cortical porosity in the regimens of 30 μg·kg^−1^ at 2/d (H: 2/d) and 6 μg·kg^−1^ at 3/d (M: 3/d; Figure 4a).

We next plotted the average cortical porosity (%) against the total amount of TPTD administration (μg·kg^−1^ per week; Figure 4b). In the ventral cortical potion, a marked increase in the cortical porosity (>15%) was observed in the regimens of 30 μg·kg^−1^ at 2/d (H: 2/d) and 6 μg·kg^−1^ at 3/d (M: 3/d), whereas other regimens showed increases of <10%. For the dorsal cortical portion, all of the regimens showed scores of <5% (Figure 4c and d). These findings suggested that the augmentation of the cortical porosity was due not merely to changes in the total dose of TPTD but also the frequency of administration. This was specifically demonstrated by the fact that, although the total amount of TPTD administered in the regimen of 30 μg·kg^−1^ at 1 day (H: 1/d) was higher than that in the regimen of 6 μg·kg^−1^ at 3 days (M: 3/d), the porosity score was <10% when compared with the vehicle controls. Furthermore, this phenomenon was demonstrated to be highly site-specific in the same bone elements of the vertebral body.

### Ventral wall-specific bone outgrowth and development of cortical porosity demonstrated by tiling fluorescence imaging

**Figure 5:**
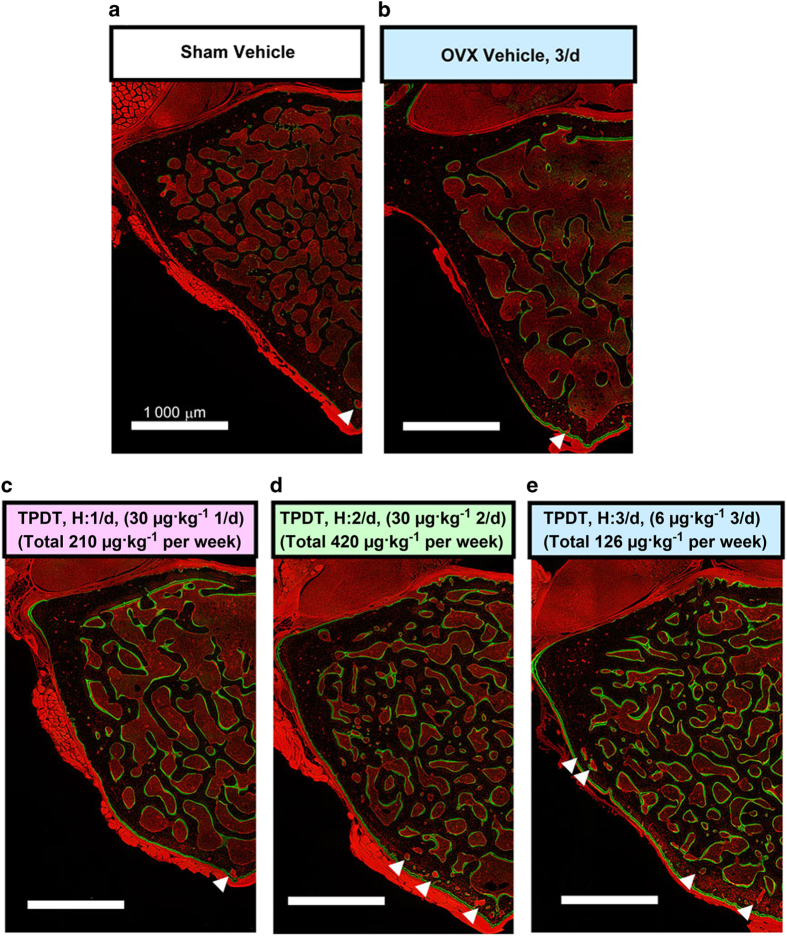
Fluorescence tiling images with deconvolution fluorescence microscopy of transverse sections of the vertebral bodies obtained from specimens in the sham vehicle group, OVX vehicle group (V: 3/d), and 30 μg·kg^−1^ at 1 day (H: 1/d), 30 μg·kg^−1^ at 2 days (H: 2/d), and 6 μg·kg^−1^ at 3 days (M: 3/d) groups (**a**–**e**, respectively). The green fluorescence signal from calcein labeling demarcates active bone formation sites, whereas the auto-fluorescence signal derived from soft tissue provides morphological information. The white arrowheads indicate obvious intracortical bone resorption. Scale bars, 1 000 μm. Dorsal to up. The corresponding bright-field images with differential interference contrast (DIC) are shown in Supplementary Figure 4.

To assess the site-specific distinction described above, we used fluorescence tiling imaging with a deconvolution fluorescence microscopy, which enables the depiction of the spatial distribution of fluorescence signals in a whole-tissue section^31^ (Figure 5). Figure 5 shows the fluorescence tiling images of LV4 for the sham vehicle control, OVX vehicle control (V: 3/d), 30 μg·kg^−1^ at 1 day (H: 1/d), 30 μg·kg^−1^ at 2 days (H: 2/d), and 6 μg·kg^−1^ at 3 days (M: 3/d) groups (Figure 5a–e, respectively). Green fluorescence signals with calcein labeling showed active bone formation in the trabecular bone and periosteal region of the ventral cortical wall, but not in the dorsal wall, at all TPTD administration levels, indicating the stimulated ventral outgrowth of the vertebral body. Red fluorescence signals by the auto-fluorescence of the soft tissue, such as muscle, periosteum, bone marrow, bone cavities encircling blood vessels, and osteocytic lacunae, clearly demarcated the spatial distribution of the unmineralized tissues. Consistent with the observations on micro-CT, the development of cortical porosity due to intracortical bone resorption was observed predominantly in the ventral cortical walls in specimens from the 30 μg·kg^−1^ at 2 days (H: 2/d) and 6 μg·kg^−1^ at 3 day (M: 3/d) groups (shown by white arrowheads). In these regimens, we further noticed a greater accumulation of red fluorescence signals derived from osteocytic lacunae in the ventral cortical wall in the dorsal wall, which was also confirmed by differential interference contrast tiling images of the corresponding specimens (Supplementary Figure 4).

### Contribution of the increased number of osteocytes to cortical porosity

**Figure 6:**
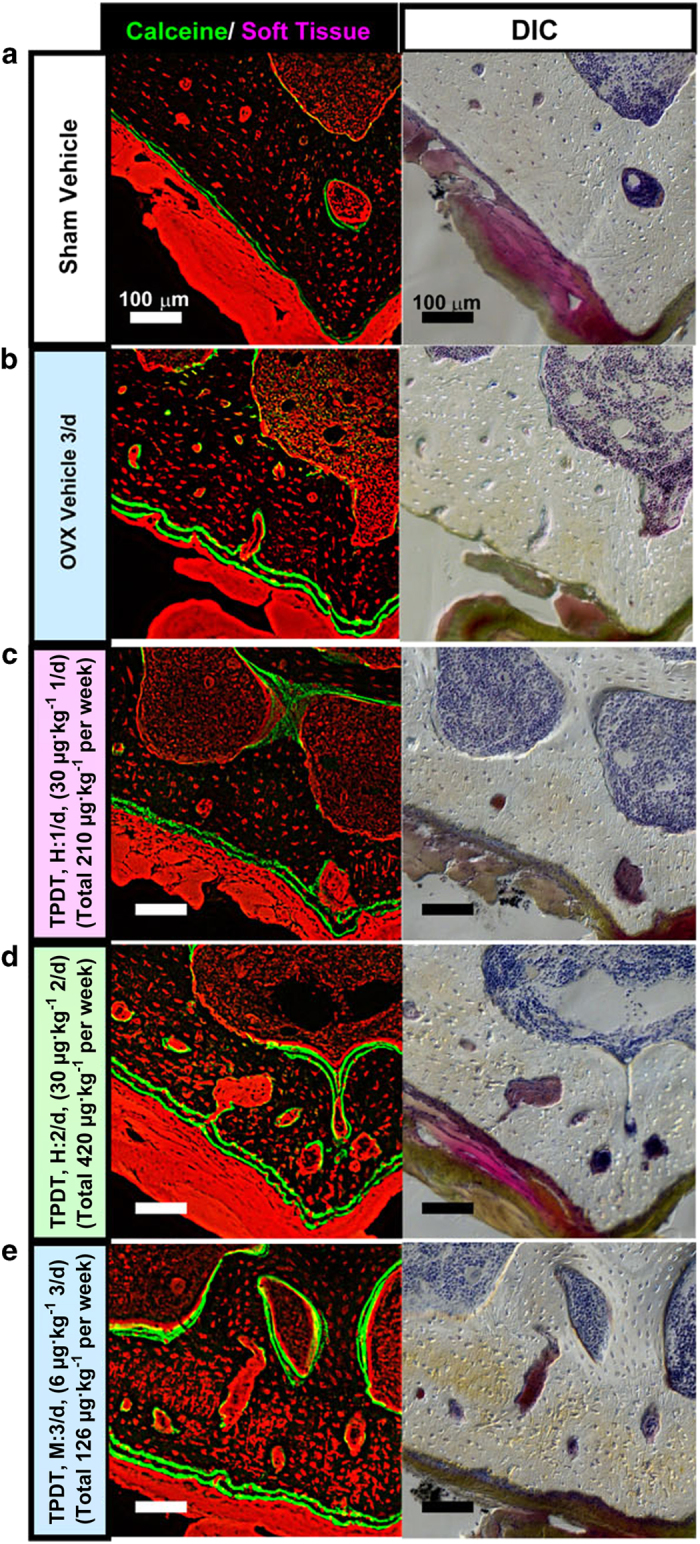
Magnified views focusing on the ventral portion of the large tiling images shown in Figure 5. The images acquired from specimens of the sham vehicle group, OVX vehicle group (V: 3/d), and 30 μg·kg^−1^ at 1 day (H: 1/d), 30 μg·kg^−1^ at 2 days (H: 2/d), and 6 μg·kg^−1^ at 3 days (M: 3/d) groups (**a**–**e**, respectively) are arranged with corresponding bright-field images with differential interference contrast (DIC). Scale bar, 100 μm. Dorsal to up.

To further assess these distinct spatial distribution patterns of osteocytic lacunae, we observed the same specimens with zoomed views of the tiling images, focusing on the ventral cortical walls (Figure 6). In specimens from the 30 μg·kg^−1^ at 2 days (H: 2/d) and 6 μg·kg^−1^ at 3 day (M: 3/d) groups, an anisotropic arrangement and increased size and number of osteocytic lacunae were obviously demonstrated. Of note, some of the osteocytic lacunae were fused, thus contributing to the cortical void formation.

**Figure 7:**
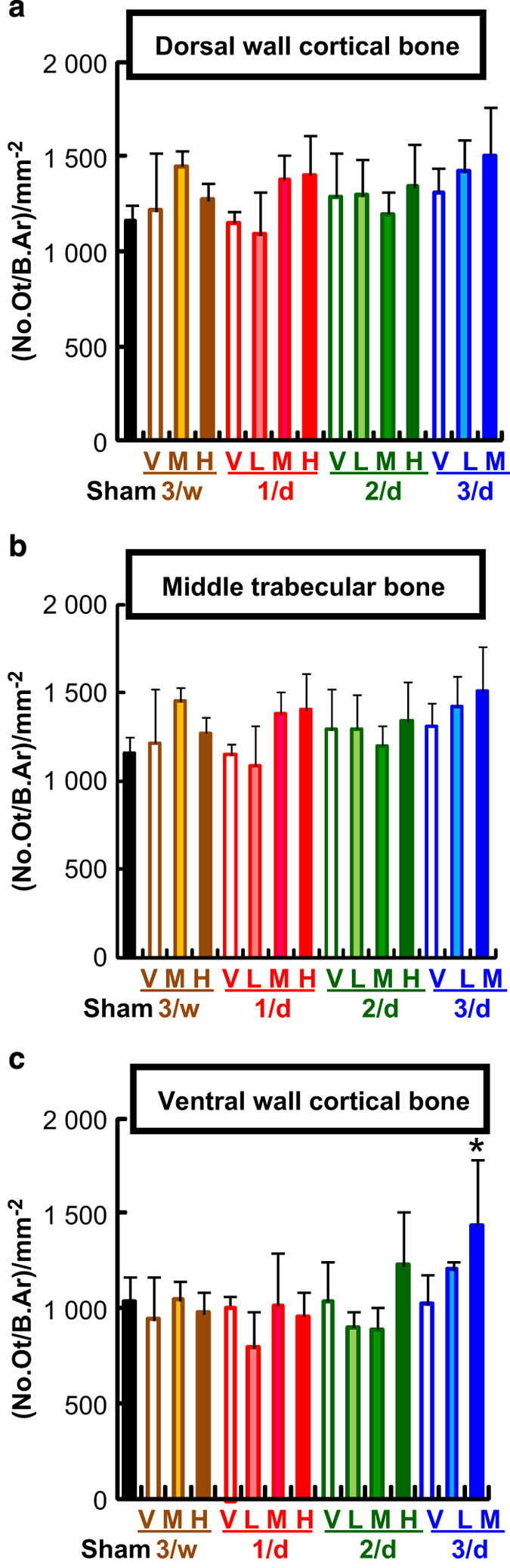
A comparison of the number of osteocytes. A histomorphometric analysis of the sagittal sections of the LV4 (**a**–**c**) parameters of dorsal cortical wall (**a**), middle trabecular area (**b**), and ventral cortical wall (**c**) are shown. The data are shown as the means+s.d. (*n*=4 or 5). *Indicates *P*<0.05 vs vehicle of each administration frequency (analysis of variance with *post hoc* Dunnett’s test). V: vehicle control, L: 1.2 μg·kg^−1^, M: 6 μg·kg^−1^, H: 30 μg·kg^−1^.

A histomorphometrical analysis demonstrated that the ventral cortical wall in specimens receiving these regimens had higher numbers of osteocytes than those in specimens from other regimens (Figure 7). Therefore, the fused lacunae might have developed due to the increased proximity of osteocytic lacunae induced by overdose or high-frequency administration of TPTD.

## Discussion

This study used a standard OVX rat model of post-menopausal osteoporosis to examine the effects of TPTD at distinct dosage and frequency of administration on BMD, mechanical properties of bone, systemic bone formation and resorption markers, and development of cortical porosity. In our regimens, doses of TPTD administered to rats were determined by pharmaco-kinetic comparisons between rats and post-menopausal women, whereas administration schedule was established by comparative analyses of the bone turnover rate of OVX rats and post-menopausal women.^27,32–35^ As a common OVX rat model to examine the pharmacological action of TPTD, we performed OVX surgery in animals at 13 weeks of age.^23–25^ In this model, a significant reduction in the BMD in the OVX group compared with that in the sham group is observed starting from 3 months after the surgery.^23,36–39^ This study monitored blood and urine samples until 7 weeks after surgery (4 weeks after the initial TPTD treatment), and dissected specimens at 7 weeks after the surgery for morphological evaluations to observe the early pharmacological actions of TPTD, especially on changes in the microarchitecture. In fact, we did not observe significant differences in the BMD, mechanical properties, average serum OC, or average cortical porosity between the sham group and OVX vehicle group, which is consistent with our previous report.^27^ Nevertheless, our analyses demonstrated that the effects of TPTD on the BMD, mechanical properties, and bone turnover were fundamentally associated with the total amount of TPTD administered, whereas the development of cortical porosity was affected by the administration frequency as well as the dosage.

Under our established regimens, the groups of 30 μg·kg^−1^ at 2 days (H: 2/d) and 6 μg·kg^−1^ at 3 day (M: 3/d) showed marked increase in the cortical porosity in the ventral portion of the vertebral body. These regimen groups also showed significant increases in the serum level of CTX as a marker of bone resorption. This correlation suggests that the development of cortical porosity strongly contributes to the systemic bone resorption marker levels in this experimental model study. Supporting this notion was our observation that the 30 μg·kg^−1^ at 2 days (H: 2/d) and 6 μg·kg^−1^ at 3 day (M: 3/d) groups did not have significantly augmented ES/BS or Oc.S/BS in the trabecular bone. This finding in our rat OVX model is consistent with those from a clinical study in elderly post-menopausal women in which increased levels of bone turnover makers were associated with higher cortical porosity and thinner cortices.^17^

The increased trabecular BMD and the development of intracortical bone resorption mentioned above phenocopies transgenic mice expressing the constitutive-active form of PTH1R (caPTH1R) driven by osteoblast- or osteocyte-specific promoters such as col1al and Dmp1, respectively.^40,41^ Therefore, high-frequency administration as well as over-dosage of TPTD may exert similar effects on bone properties to that of constitutively activated PTH1R-mediated signaling in osteoblast–osteocyte lineages.

The development of cortical porosity in these regimens was preferentially observed in the ventral cortical wall of vertebrae, suggesting that the local bone environment and/or site-specific bone architecture could be a critical rationale for developing cortical porosity, as well as the regimen of TPTD administration. Deconvolution fluorescence tiling imaging demonstrated that TPTD administration preferentially stimulated the outgrowth of the ventral cortical bone, in which the development of cortical porosity was also predominantly observed. As the anabolic effect of TPTD administration is reportedly associated with mechanical loading to the bone,^42,43^ local distinctions in mechanical loading are likely to affect the site-specific bone growth and the development of intracortical bone resorption.

Our findings further showed that the increased number and proximity of osteocytic lacunae occasionally forming fused lacunae induced by TPTD can be a critical element involved in developing cortical porosity. Several studies have suggested that distinct mechanical loading at local bone sites affects the microarchitecture and functions of osteocyte-mediated cellular networks, thereby contributing to the locally different properties of bone remodeling that respond to local and systemic regulations.^44,45^ Therefore, site-specific increases in the osteocytic lacunae induced by TPTD administration may also be related to local distinctions in mechanical loading.

The molecules that regulate the transition from osteoblasts to osteocytes are largely unknown.^46^ In addition, how the ratio of this transition from a given population of bone-forming osteoblasts is controlled is also unknown. Morphologically, the transition to osteocytes involves dynamic changes in cellular shape, such as the extension of the cellular process and reduction in cell body size.^47,48^ Generally, the reduction in cell size is tightly associated with the reduction in cytoplasmic organelles such as mitochondria and the endoplasmic reticulum.^49^ Therefore, the local vasculature that supplies oxygen to the adjacent cells may affect the rate of osteocyte differentiation as well as osteocyte function. However, how the mechanical loading and stimulated rate of bone formation by TPTD administration affect the formation rate of osteocytes remains to be clarified.

The increased size of the osteocytic lacunae observed in bone sites that develop cortical porosity suggests the involvement of osteocytic osteolysis in forming the fused lacunae and cortical porosity.^50,51^ Qing *et al*^52^ reported that tartrate-resistant acid phosphatase and cathepsin K were involved in osteocytic osteolysis. They also reported that no osteocytic remodeling was observed with experimental unloading. These present and previous findings suggest that bone site-specific distinction in mechanical loading may be a critical factor for the induction of osteocytic osteolysis, which could contribute to the development of intracortical porosity.

PTH binding to PTH1R preferentially simulates endosome-mediated long cAMP signaling, whereas PTHrP prefers to stimulate canonical short cAMP signaling.^9^ The stabilized PTH analog D6 exhibits a longer half-life than native PTH(1–34) in the blood stream, inducing an increase in the blood Ca^2+^ level that persists longer than that of PTH(1–34).^10^ This report indicates that a prolonged increase in the blood PTH level is closely associated with the catabolic effect of PTH. The other PTH analog, M-PTH(1–34), selectively binds to the R0 state PTH1R which preferentially stimulates the endosome-mediated long signal.^11^ The half-life of this analog in the blood stream is much shorter than that of PTH(1–34). However, it stimulates a longer calcemic response than does PTH(1–34), which is similar to the effects of D6. This observation is explained by the fact that M-PTH(1–34) binds to a specific PTH1R conformation (the R0 state) in a pseudo-irreversible manner. These findings together demonstrate that continuous or prolonged stimulation of the endosome-mediated long cAMP signaling by PTH exerts a catabolic effect on bone metabolism.

On the basis of our series of comparative studies of pharmaco-dynamics and bone turnover rate between OVX rats and post-menopausal women, the single administration dose of 6 μg·kg^−1^ used in this study corresponds to a dose two times higher than that of 56.5 μg once-weekly administration used in clinical setting based on the areas under the concentration–time curves,^53–55^ whereas once daily and three times weekly administrations in rats are estimated to be equivalent to once-weekly administration in post-menopausal women.^27,32–35^ Therefore, our observation in this work suggests that over-frequency or over-doses of TPTD administration in clinical situations may be a causative of cortical porosity, whose development is associated with local distinction in mechanical properties of bone sites. Although these findings were obtained from experiments with young adult rats, the same regimens may also cause cortical porosity in older rats, as our previous study has demonstrated the effects of TPTD in aged OVX rats.^24^ Given that clinical evidence has indicated the relevance of cortical porosity and thinner cortices as determinants of bone fragility and fracture risk,^15–18^ our findings here underscore the importance of the TPTD treatment regimen in inhibiting the development of cortical porosity and augmentation of the BMD. It also should be noted that, unlike post-menopausal women, the young adult rats used in this work were still growing, which is a distinct situation in the clinical administration of TPTD; however, the vertebral bodies analyzed in this work did not show obvious outgrowth, as evidenced by the observations in the sham/vehicle group. Therefore, further studies in animals with bone remodeling, such as rabbits, should be informative.

In the present study, however, we observed that an increased administration frequency as well as dosage of TPTD-induced cortical porosity was associated with an increase in the levels of systemic bone resorption markers. This finding was likely due to the fact that a prolonged or persistent PTH level at a given threshold in blood stream constitutively stimulated the long cAMP signaling, thereby causing cortical porosity that involved site-specific intracortical bone resorption, an increased number of osteocytes, and possible osteocytic osteolysis.

In conclusion, our regimens of TPTD in a standard OVX rat model of post-menopausal osteoporosis showed that the development of cortical porosity was associated with high administration frequency as well as the dosage, which is plausibly explained by constitutively activated endosome-mediated intracellular signaling by TPTD in osteoblast-lineage cells. Furthermore, the combined analysis of three-dimensional micro-CT and successive deconvolution fluorescence tiling imaging in this study enabled us to manifest the spatial development of cortical porosity in a consistent manner from the whole-tissue to cellular level. Our observations suggested that the distinction of the mechanical properties at local bone sites was a critical factor for developing cortical porosity. The association between the duration of cAMP signaling and the downstream responses that promote bone anabolism and catabolism, which could also unravel the PTH paradox of intermittent versus continuous administration, should be investigated in future studies. These molecular pathways may be affected by mechanical loading and unloading to bone cells. Further investigation and consideration are still needed to extrapolate clinical situation from data obtained from animal models in terms of the development of cortical porosity.

## Figures and Tables

**Table 1:** The regimen settings of teriparatide [human PTH(1–34), TPTD] administration in ovariectomized rats

Group		Operation	Treatment	Dose		Frequency	*n*
				(μg·kg^−1^)	(μg·kg^−1^ per week)		
1	Sham	Sham	Vehicle	—	—	3/week	5
2	W3V	OVX	Vehicle	—	—	3/week	5
3	W3M	OVX	TPTD	6	18	3/week	5
4	W3H	OVX	TPTD	30	90	3/week	5
5	D1V	OVX	Vehicle	—	—	1/day	5
6	D1L	OVX	TPTD	1.2	8.4	1/day	5
7	D1M	OVX	TPTD	6	42	1/day	5
8	D1H	OVX	TPTD	30	210	1/day	5
9	D2V	OVX	Vehicle	—	—	2/day	5
10	D2L	OVX	TPTD	1.2	16.8	2/day	5
11	D2M	OVX	TPTD	6	84	2/day	5
12	D2H	OVX	TPTD	30	420	2/day	5
13	D3V	OVX	Vehicle	—	—	3/day	5
14	D3L	OVX	TPTD	1.2	25.2	3/day	5
15	D3M	OVX	TPTD	6	126	3/day	5

Abbreviations: TPTD, teriparatide; OVX, ovariectomized.

In total, 15 experimental groups were established with four administration schedules: three times a week (3/w), once a day (1/d), twice a day (2/d), and three times a day (3/d), and four distinct TPTD doses of 0 (vehicle: V), 1.2 (low: L), 6 (medium: M), and 30 μg·kg^−1^ (high: H) for 4 weeks.
