# Supplementary figures and images for: Administration frequency as well as dosage of PTH are associated with development of cortical porosity in ovariectomized rats
Source: Bone Res. 2017 Apr 25;5:17002–. doi: 10.1038/boneres.2017.2 (PMC5405404; doi:10.1038/boneres.2017.2)

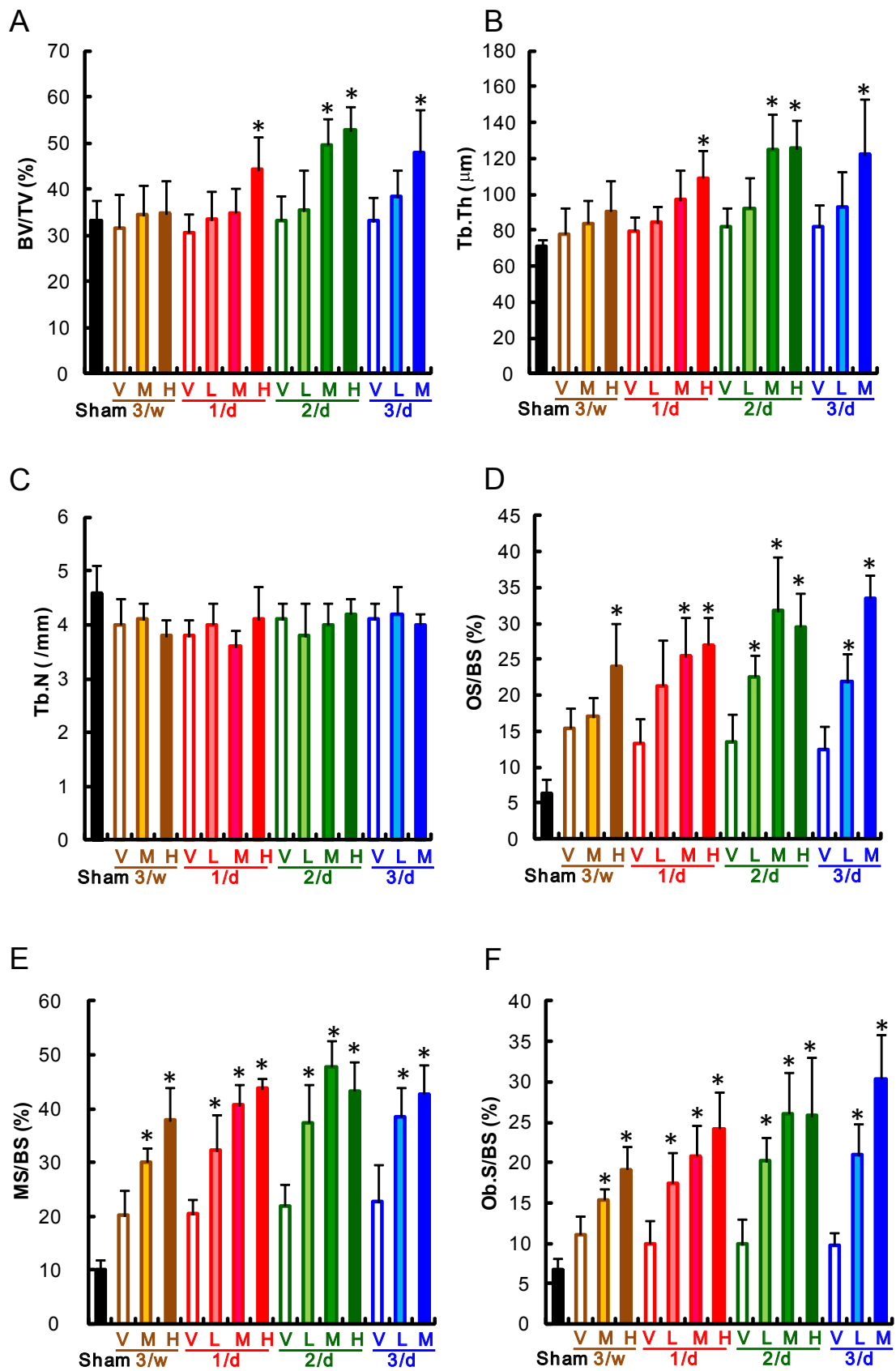

Supplement: Supplementary Figure 1 [file boneres20172-s1.pdf]

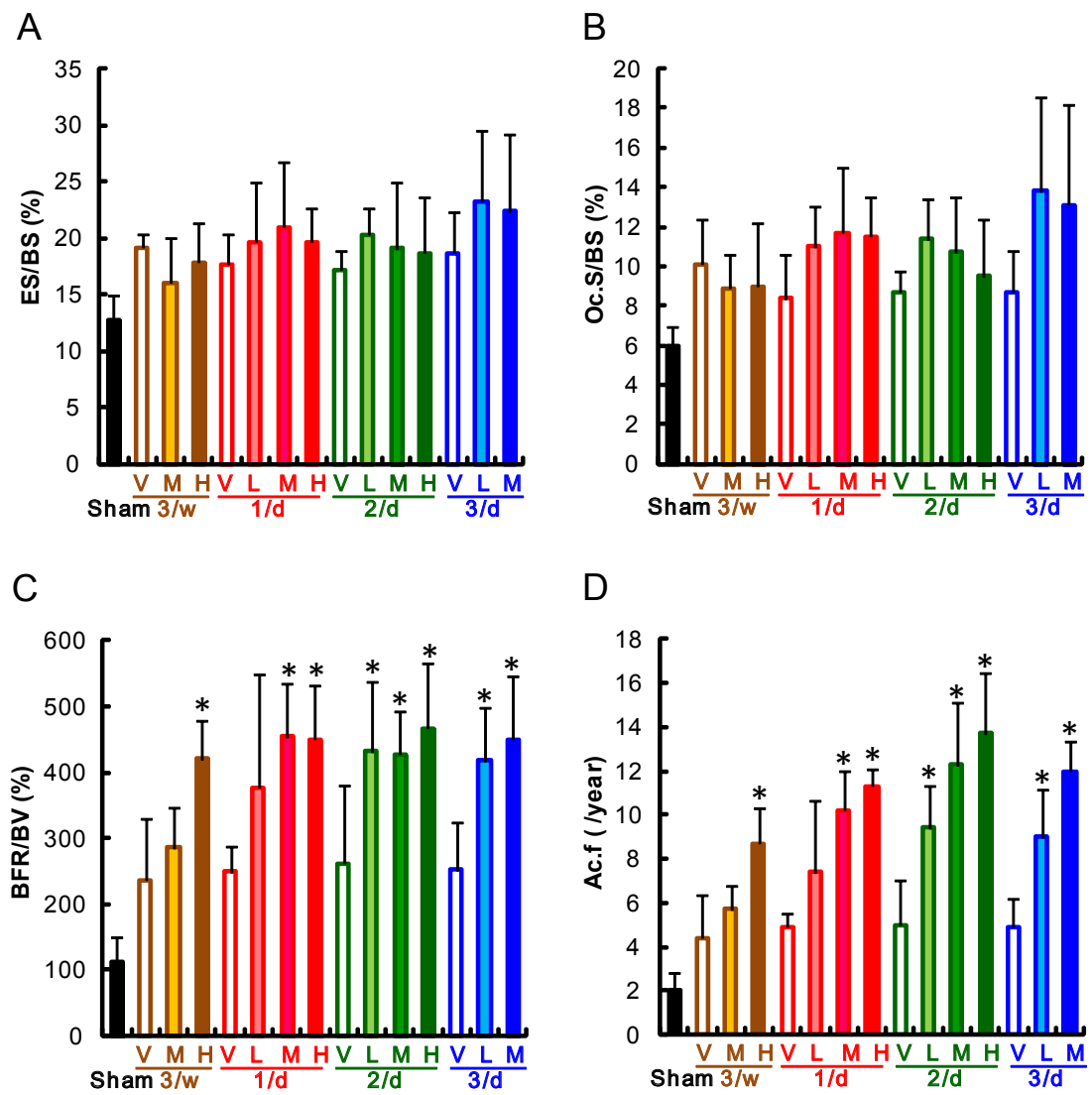

Takakura et al., Supplementary Figure 2

Supplement: Supplementary Figure 2 [file boneres20172-s2.pdf]

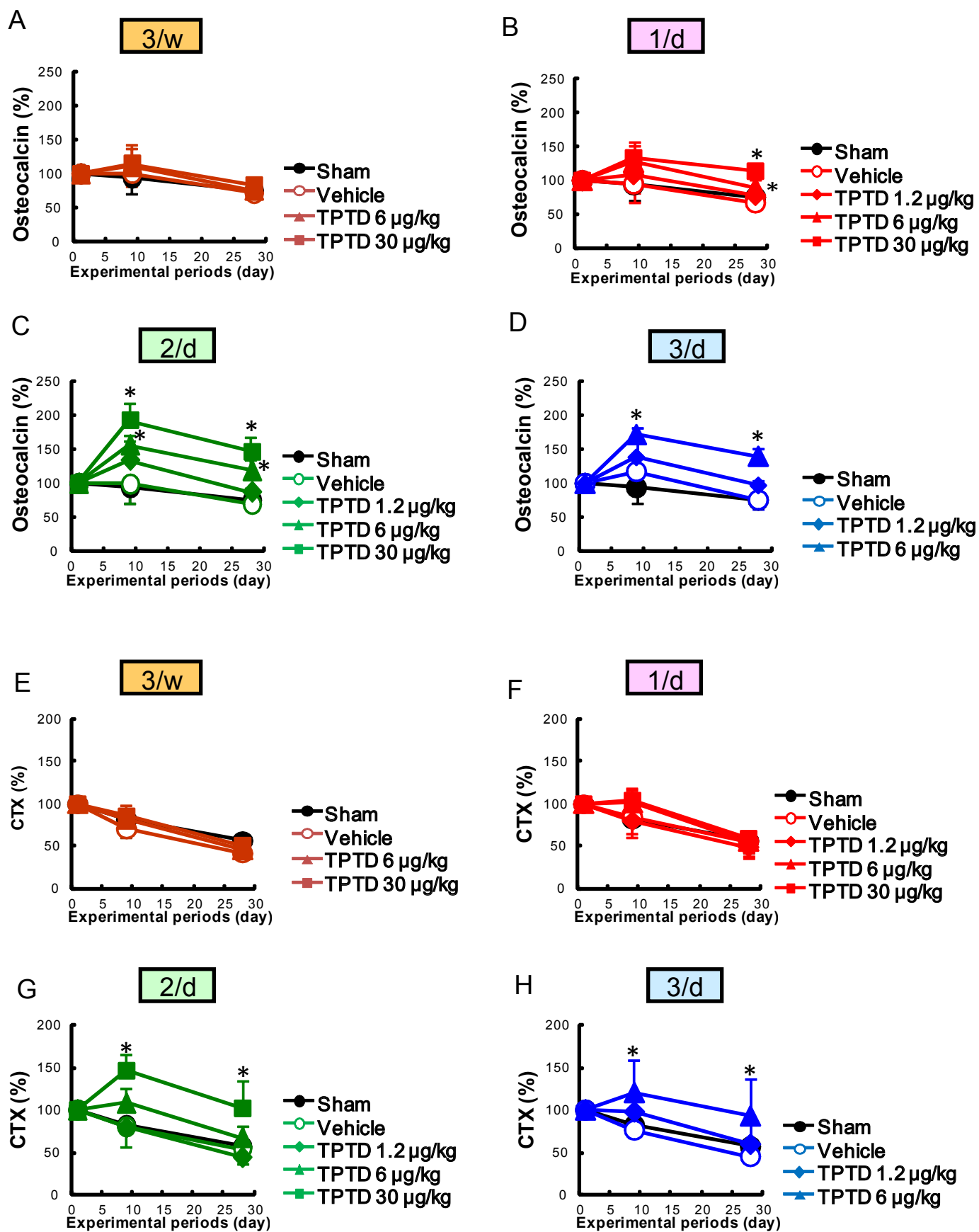

Takakura et al., Supplementary Figure 3

Supplement: Supplementary Figure 3 [file boneres20172-s3.pdf]

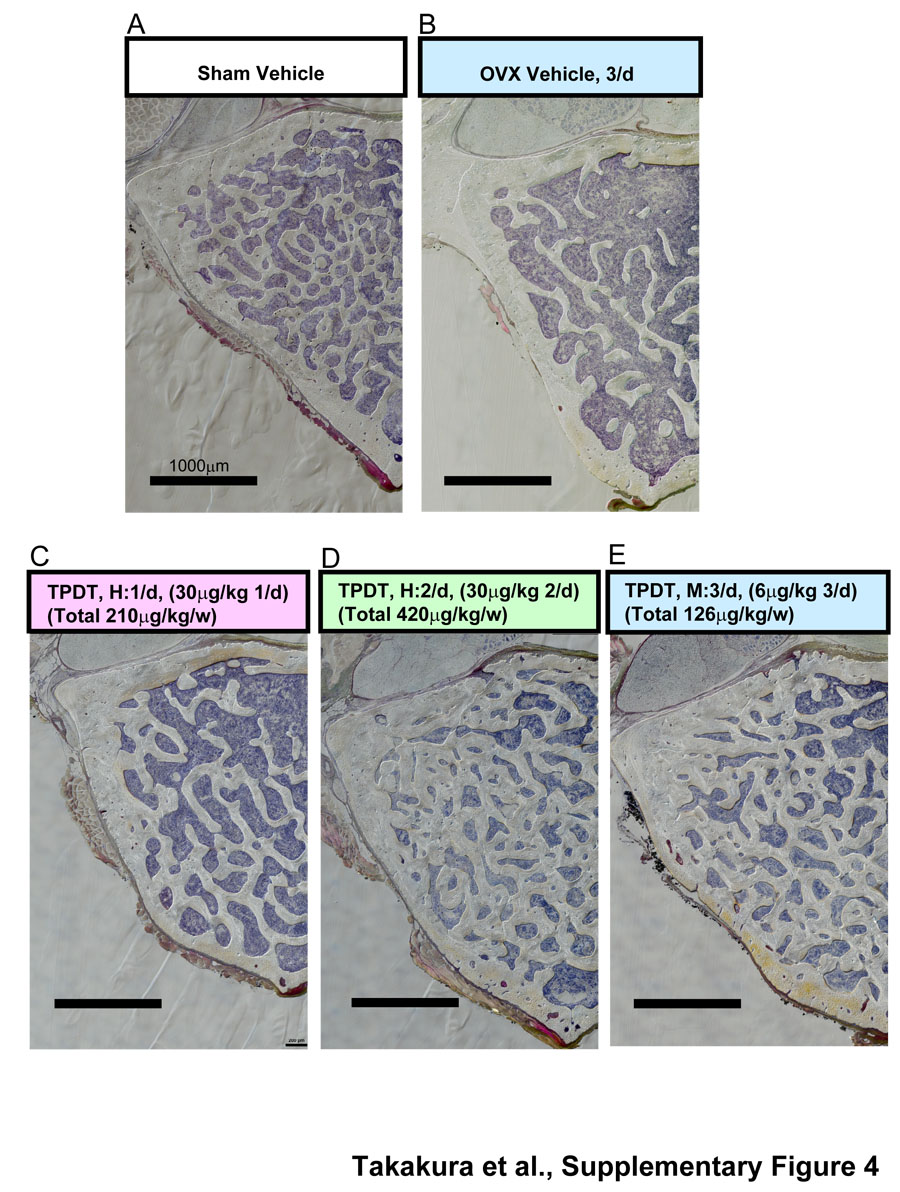

Supplement: Supplementary Figure 4 [file boneres20172-s4.jpg]
